# Serum Chemerin Concentrations and Tissue Immunoreactivity in Colorectal Adenoma and Colorectal Cancer: An Exploratory Case–Control Study

**DOI:** 10.3390/diagnostics16162589

**Published:** 2026-08-16

**Authors:** Piotr Szredzki, Aleksandra Szredzka, Anna Belina, Maria Marlicz, Mikołaj Podlasek, Paweł Guzik, Tomasz Góra, Anastasios Koulaouzidis, Wojciech Marlicz, Karolina Skonieczna-Żydecka, Michał Kukla

**Affiliations:** 1 Department of General Surgery, University of Rzeszów, 35-959 Rzeszów, Poland; piotrszredzki@gmail.com (P.S.); aniakrajewicz@gmail.com (A.B.); 2 Endoscopy Department, Municipal Hospital in Rzeszów, 35-241 Rzeszów, Poland; 3 Department of Immunology, Medical University of Warsaw, 02-091 Warsaw, Poland; aleksandra.szredzka@gmail.com; 4 Department of Biochemical Sciences, Pomeranian Medical University, 70-204 Szczecin, Poland; mariamarlicz@gmail.com (M.M.);; 5 Department of Vascular Surgery, St. Padre Pio Provincial Hospital in Przemyśl, 37-700 Przemyśl, Poland; podlasekmi@gmail.com; 6 Department of General Gynecology and Obstetrics, University of Rzeszów, 35-959 Rzeszów, Poland; pawelguzik@gmail.com (P.G.); mindir@gmail.com (T.G.); 7 Department of Gastroenterology, Pomeranian Medical University, 70-204 Szczecin, Poland; marlicz@hotmail.com; 8 Department of Gastroenterology and Hepatology, Jagiellonian University Medical College, 30-688 Kraków, Poland; michal.kukla@uj.edu.pl

**Keywords:** chemerin, colorectal cancer, colorectal adenoma, serrated lesion, adipokine, exploratory biomarker study, immunohistochemistry

## Abstract

**Background/Objectives:** Chemerin is an adipokine implicated in metabolic regulation, inflammation and cancer biology, but its value as a circulating biomarker in colorectal neoplasia remains uncertain. We investigated serum chemerin concentrations in patients with colorectal cancer (CRC), patients with colorectal polyps and colonoscopy-negative controls, and explored chemerin immunoreactivity in available polyp and tumour tissue. **Methods:** This exploratory observational case–control study included 41 patients with CRC, 20 patients with colorectal polyps and 29 colonoscopy-negative controls. Serum chemerin was measured by ELISA. Tissue specimens underwent routine histopathology and qualitative immunohistochemical staining for chemerin. Between-group comparisons were performed using non-parametric tests; subgroup analyses were exploratory and unadjusted. **Results:** Serum chemerin concentrations did not differ significantly between CRC and controls (median 144.0 vs. 135.7 ng/mL; *p* = 0.41), CRC and polyp groups (144.0 vs. 97.4 ng/mL; *p* = 0.24), or polyp and control groups (97.4 vs. 135.7 ng/mL; *p* = 0.94). Chemerin immunostaining was absent in CRC tissue (0/41) and conventional adenomatous polyps (0/15), whereas all available hyperplastic/serrated lesions showed epithelial cytoplasmic and/or stromal immunoreactivity (5/5); in lesions containing dysplasia, dysplastic glands showed no detectable staining. In controls, higher chemerin concentrations were associated with hyperglycaemia, hypertension, body weight and bilirubin, but these exploratory findings were not adjusted for multiplicity or metabolic confounding. **Conclusions:** In this exploratory cohort, serum chemerin did not discriminate CRC or colorectal adenoma from colonoscopy-negative controls. Together with the absence of staining in CRC and conventional adenomas, the findings argue against chemerin as a standalone circulating or commonly expressed tissue biomarker for these lesions under the assay conditions used. Immunoreactivity in the non-dysplastic epithelial and/or stromal compartments of hyperplastic/serrated lesions remains preliminary and requires prospective confirmation with standardised pre-analytics, quantitative pathology scoring and adjustment for metabolic confounders.

## 1. Introduction

### 1.1. Aetiology and Epidemiology of Colorectal Polyps and Colorectal Cancer

Adenomatous colorectal polyps are neoplastic epithelial lesions characterised by glandular proliferation and dysplasia. Conventional adenomas are usually classified as tubular, tubulovillous or villous, and their malignant potential is influenced by size, histological architecture and degree of dysplasia. Serrated colorectal lesions represent a distinct pathway to colorectal cancer and include hyperplastic polyps, sessile serrated lesions and traditional serrated adenomas [1,2]. Their biological behaviour is heterogeneous: many small distal hyperplastic polyps have limited malignant potential, whereas larger proximal serrated lesions, particularly those with dysplasia, may carry clinically important neoplastic risk [2]. For this reason, conventional adenomas and serrated/hyperplastic lesions should not be treated as a single biological category when tissue biomarker findings are interpreted.

### 1.2. Impact of Abdominal Obesity on Cancer Risk

Obesity and metabolic dysfunction are established risk factors for several gastrointestinal malignancies, including colorectal cancer [3,4]. The mechanisms linking excess adiposity with carcinogenesis are complex and include chronic low-grade inflammation, insulin resistance, altered insulin-like growth factor signalling, adipokine dysregulation, metabolic dysfunction-associated steatotic liver disease and changes in the intestinal microbiota [5,6,7,8,9,10]. These mechanisms are relevant to the present study because chemerin concentrations are influenced by metabolic phenotype, including adiposity, glucose status and blood pressure [11,12,13].

### 1.3. Chemerin

Chemerin is produced predominantly by the liver and white adipose tissue and is involved in immune cell recruitment, inflammation, adipogenesis, glucose metabolism and angiogenesis [11,12,14,15,16]. Increased circulating chemerin concentrations have been reported in several inflammatory and metabolic conditions, including chronic liver disease, non-alcoholic fatty liver disease, obesity-related metabolic dysfunction and metabolic syndrome [8,11,12,13,14]. These associations make chemerin biologically plausible as a candidate marker linking metabolic inflammation and colorectal neoplasia, but they also make it vulnerable to confounding by body composition, glucose status, blood pressure, medication use, age and sex [11,12,13].

The biological role of chemerin in cancer is context-dependent. Experimental and clinical studies have suggested both pro-inflammatory and anti-inflammatory effects [15,16], and published studies on CRC have reported inconsistent associations between circulating chemerin and cancer presence, tumour stage, sex and tumour location [17,18,19]. Therefore, small unadjusted biomarker studies should be interpreted cautiously and framed as exploratory unless supported by adequate sample size, detailed pathological characterisation and adjustment for metabolic confounders.

The rationale for the present study was to examine whether serum chemerin differs across colonoscopy-defined clinical groups and whether tissue chemerin immunoreactivity is detectable in colorectal neoplastic and pre-neoplastic lesions, given prior reports linking chemerin to CRC risk, CRC presence and tumour stage [17,18,19]. Because the cohort is modest and the tissue assessment is qualitative, the study is best positioned as an exploratory case–control analysis rather than a definitive biomarker validation study. The study was not designed to establish a cellular biochemical pathway or causal mechanism; it addressed the narrower diagnostic question of whether circulating concentrations or tissue immunoreactivity differed across the predefined clinical and pathological groups.

The primary objective was to compare serum chemerin concentrations among patients with CRC, patients with colorectal polyps and colonoscopy-negative controls. Secondary exploratory objectives were to describe chemerin immunoreactivity in available polyp and tumour tissue and to examine unadjusted associations between serum chemerin and selected metabolic parameters.

## 2. Materials and Methods

### 2.1. Study Design and Participants

This was an exploratory, observational, colonoscopy-defined case–control study of adults undergoing clinically indicated colonoscopy. Participants were allocated after colonoscopy and histopathological assessment into one of three mutually exclusive groups: (1) patients with histologically confirmed colorectal adenocarcinoma; (2) patients with colorectal polyps and no colorectal cancer; and (3) colonoscopy-negative controls, defined as subjects in whom colonoscopy did not identify colorectal cancer or colorectal polyps. The cohort was drawn from symptomatic or clinically indicated colonoscopy referrals, including anaemia, altered bowel habit, gastrointestinal transit symptoms and positive faecal immunochemical testing. A precise patient-level breakdown of referral indication was not available.

### 2.2. Eligibility Criteria

The inclusion criteria were age 18–80 years; clinically indicated colonoscopy; availability of serum chemerin measurement; provision of informed consent; and assignment to one of the three study groups on the basis of colonoscopy and histopathology. The group-specific inclusion criteria were as follows: the CRC group required histologically confirmed colorectal adenocarcinoma; the polyp group required histologically confirmed colorectal polyp tissue without colorectal cancer; and the control group required a colonoscopy negative for colorectal cancer and colorectal polyps. The exclusion criteria were inflammatory bowel disease, uncontrolled diabetes, uncontrolled renal failure, hyperthyroidism or hypothyroidism, advanced heart failure (NYHA class III–IV), autoimmune disease, hepatitis B or C virus infection, alcoholism or active substance abuse, history or presence of another malignancy, depression, current or previous psychotic disorder, active tuberculosis, bronchial asthma, chronic obstructive pulmonary disease, age below 18 or above 80 years, and lack of consent.

### 2.3. Outcomes

The primary outcome was the distribution of serum chemerin concentration across the three colonoscopy-defined groups (CRC, colorectal polyp and colonoscopy-negative control), reported as median and interquartile range and compared using unadjusted non-parametric tests. Secondary outcomes were qualitative chemerin immunoreactivity in available colorectal adenocarcinoma, conventional adenomatous polyp and hyperplastic/serrated lesion tissue, and descriptive clinicopathological characterisation of the CRC and polyp groups. Exploratory outcomes were unadjusted associations between serum chemerin and selected metabolic, anthropometric and biochemical variables, including glucose status, hypertension status, body weight, BMI and available laboratory parameters.

### 2.4. Variables Collected and Measurements

The following variables were collected where available: age, sex, body weight, BMI, systolic and diastolic blood pressure, hypertension status, antihypertensive treatment, hyperglycaemia/diabetes status, statin or other lipid-lowering therapy, glucose, total cholesterol, LDL cholesterol, HDL cholesterol, triglycerides, alanine aminotransferase, aspartate aminotransferase, gamma-glutamyl transferase, alkaline phosphatase, bilirubin, creatinine, iron, INR, APTT and complete blood count. Colonoscopy and histopathology variables included study-group assignment, colorectal tumour location, AJCC 8th edition TNM stage, tumour grade, polyp anatomical location, polyp histological subtype, polyp size range, dysplasia grade and qualitative chemerin immunohistochemistry status. Diabetes and hyperglycaemia could not be separated reliably for all participants and were therefore analysed as a combined variable.

Serum chemerin concentrations were measured using a commercially available human chemerin ELISA kit (BioVendor, Brno, Czech Republic; catalogue no. RD191136200R). Assay sensitivity was 0.1 ng/mL, intra-assay variability was 6.0%, and inter-assay variability was 7.6%. Absorbance was measured using a microplate reader. Blood was collected before surgery in CRC patients and before polyp removal in patients with polyps. Biochemical blood tests were performed in the analytical laboratory of the Municipal Hospital in Rzeszów and the Diagnostics Laboratory in Rzeszów using standard laboratory methods. Beyond the timing of collection relative to surgery or polyp removal, detailed information on fasting status, collection tube type, clotting or centrifugation intervals, storage duration, and freeze–thaw cycles was not available for the present analysis.

### 2.5. Histopathology and Immunohistochemistry

Tissue sections from colorectal tumours and polyps were processed using routine histopathological techniques and stained with haematoxylin and eosin for standard pathological evaluation. Immunohistochemical analysis for chemerin was performed using monoclonal antibodies (BioVendor), with visualisation using the BrightVision plus Poly-AP-Anti Ms/Rb/Rt IgG detection system (ImmunoLogic, Waltham, MA, USA). Microscopic assessment was performed using a Nikon Eclipse 80i light microscope (Nikon Corporation, Tokio, Japan) at 100× and 200× magnification. Chemerin staining was assessed qualitatively as present or absent, with descriptive recording of epithelial, stromal and dysplastic-glandular staining patterns where visible. No quantitative or semi-quantitative immunohistochemical scoring system was applied. Colonoscopies were conducted using the Exera III endoscopic system (Olympus, Tokyo, Japan).

### 2.6. Statistical Analysis

Statistical analyses were performed using Statistica v. 7.1 PL (StatSoft, Tulsa, OK, USA) and MedCalc Statistical Software v. 14.10.2 (MedCalc Software bvba, Ostend, Belgium). Data distribution was assessed using the Shapiro–Wilk test. Because the data did not follow a normal distribution, continuous variables were summarised as medians and interquartile ranges and compared using Mann–Whitney U tests for pairwise comparisons. Categorical variables were summarised as counts and percentages and checked using chi-square tests where appropriate. Statistical significance was set at *p* < 0.05. No formal a priori sample-size calculation was performed. No multivariable adjustment for metabolic confounders and no correction for multiple comparisons were performed; therefore, subgroup and exploratory *p*-values are nominal and should be interpreted cautiously. For the tissue immunohistochemistry proportions, two-sided 95% exact binomial (Clopper–Pearson) confidence intervals were calculated post hoc to describe the precision of the observed positivity rates.

### 2.7. Reporting and Ethics

The study is reported in accordance with the STROBE framework for observational studies [20]. The study was conducted in accordance with the Declaration of Helsinki and approved by the Bioethics Committee (decision no. 18/B/2019, issued on 31 January 2019). Informed consent was obtained from all participants.

## 3. Results

A total of 90 participants were included in the final analysis: 41 patients with CRC, 20 patients with colorectal polyps and 29 colonoscopy-negative controls. The cohort included 43 women and 47 men. The baseline characteristics are shown in Table 1.

Additional comparisons of categorical baseline variables showed no significant group differences in sex distribution (chi-square *p* = 0.462), hypertension (*p* = 0.895), hyperglycaemia/diabetes status (*p* = 0.769), or statin/lipid-lowering therapy (*p* = 0.416). These checks do not replace adjusted modelling of serum chemerin.

**Figure 1:**
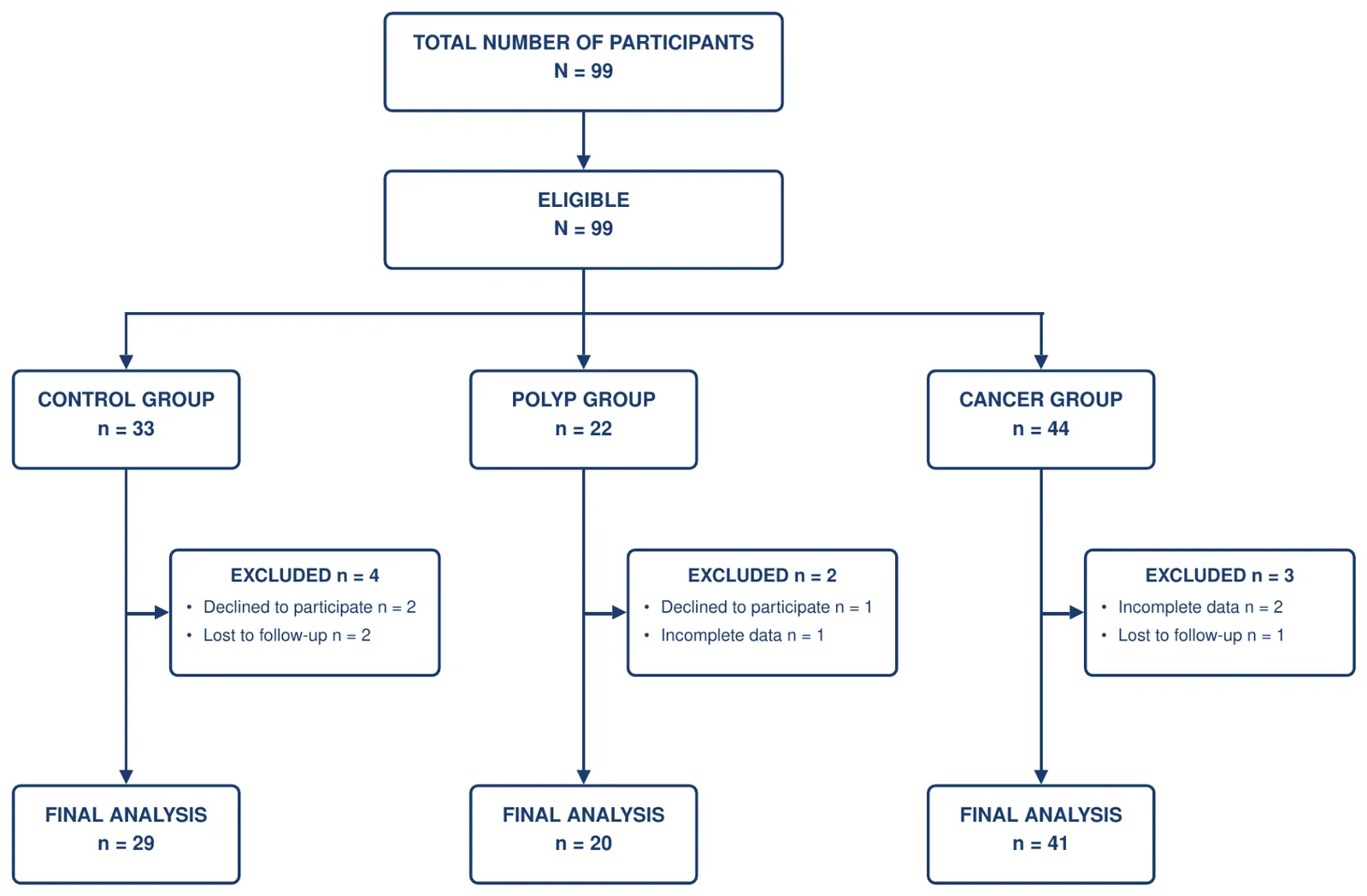
Participant flow diagram.

The participant flow is shown in Figure 1.

CRC location and AJCC 8th edition TNM stage distribution are summarised in Table 2. Staging was based on pathological diagnosis for the primary tumour and nodal status, with the M category based on imaging tests.

Polyp characteristics are summarised in Table 3, including anatomical location, histological subtype and size range.

Serum chemerin concentrations did not differ significantly among the three clinical groups in unadjusted pairwise exploratory comparisons. Median chemerin concentration was 144.0 ng/mL in the CRC group, 97.4 ng/mL in the polyp group and 135.7 ng/mL in controls. Pairwise comparisons were non-significant: CRC versus polyp group, *p* = 0.24; CRC versus controls, *p* = 0.41; and polyp group versus controls, *p* = 0.94 [Table 4]. These results should be interpreted as unadjusted exploratory comparisons rather than definitive evidence of no association.

Exploratory subgroup analyses within the CRC group did not show statistically significant differences in serum chemerin by histological grade (G1 vs. G2, *p* = 0.32; G2 vs. G3, *p* = 1.00; G1 vs. G3, *p* = 0.70). In the polyp group, median chemerin concentration was numerically lower among patients with two or more polyps than among those with a single polyp (22.5 vs. 181.9 ng/mL; *p* = 0.06), but this comparison was based on only 20 patients and should not be interpreted as evidence of a biological association between chemerin and polyp burden.

Exploratory analyses by glucose status showed higher serum chemerin concentrations among controls with elevated glucose compared with controls with normal glucose (*p* = 0.009). Exploratory analyses by blood pressure status showed higher chemerin concentrations among hypertensive controls than normotensive controls (*p* = 0.002), whereas the corresponding comparison in the polyp group was non-significant. Because no multiplicity correction or multivariable adjustment was performed, these findings should be interpreted as metabolic associations within controls rather than disease-specific effects. No robust disease-specific associations were identified between chemerin concentration and the analysed laboratory or anthropometric variables in patients with CRC or colorectal polyps.

### Immunohistochemistry

**Figure 2:**
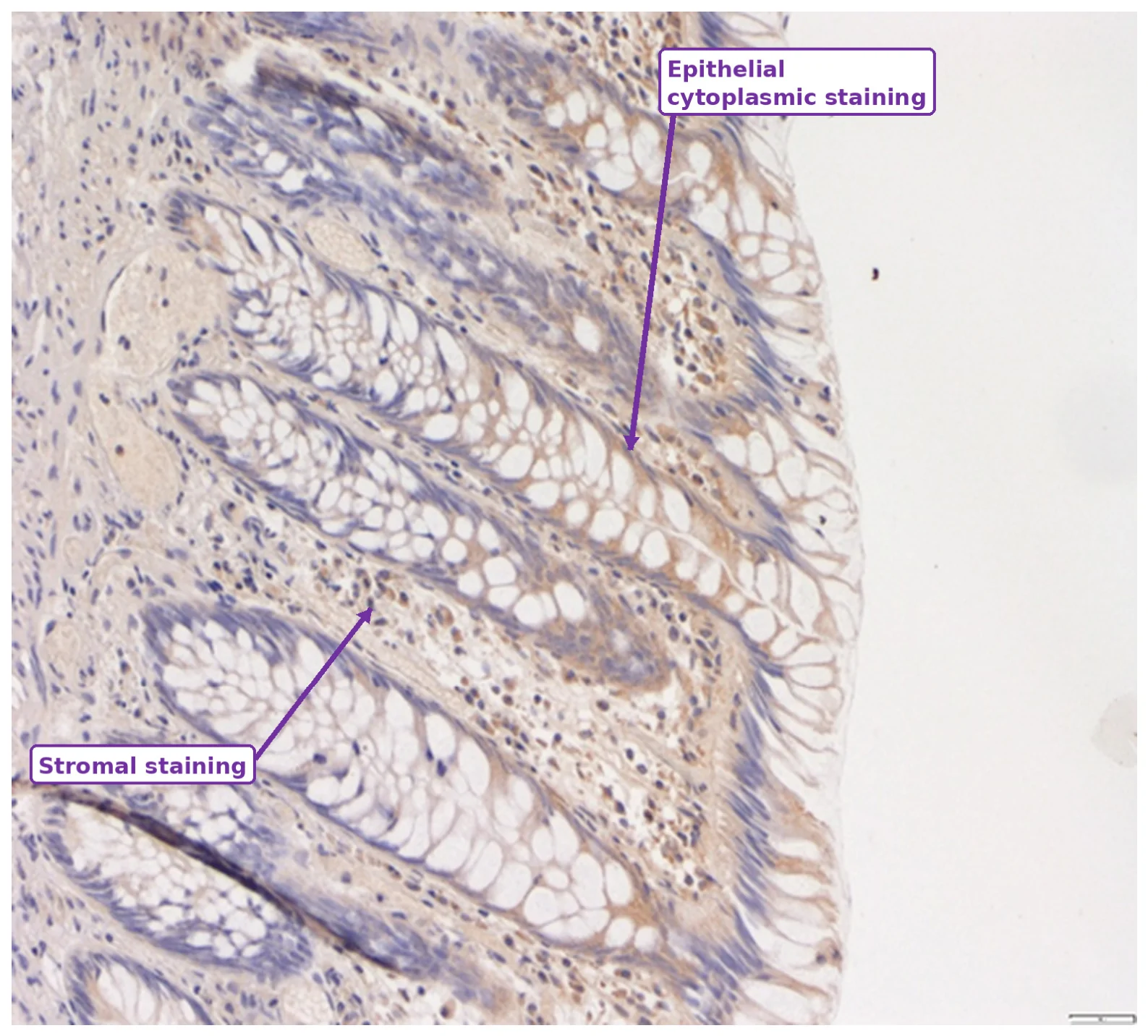
Hyperplastic/serrated lesion showing epithelial cytoplasmic and stromal chemerin immunoreactivity. Purple arrows identify epithelial cytoplasmic staining and stromal staining (Magnification 100×).

**Figure 3:**
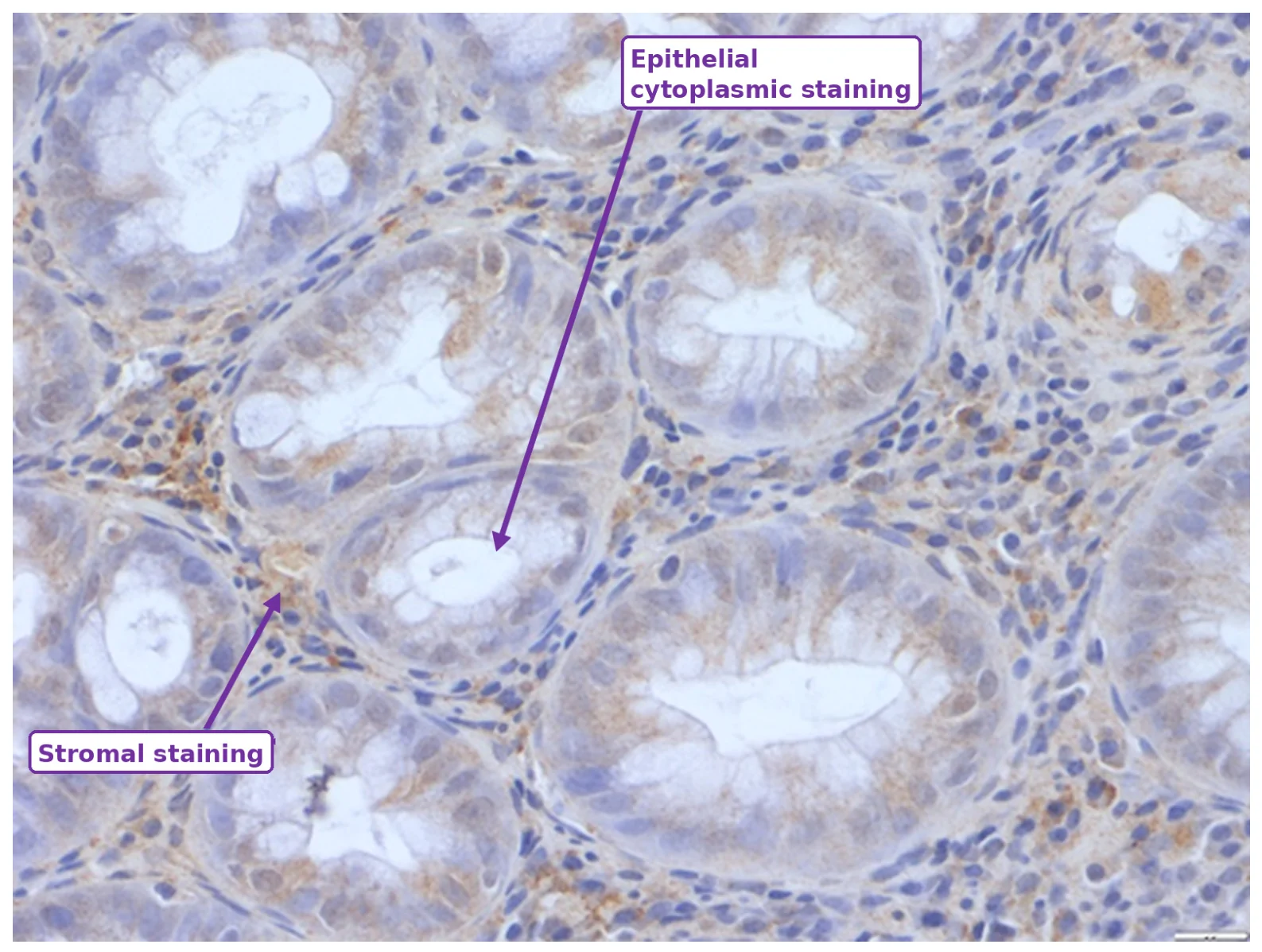
Hyperplastic/serrated lesion showing epithelial cytoplasmic and stromal chemerin immunoreactivity. Purple arrows identify epithelial cytoplasmic staining and stromal staining (Magnification 100×).

**Figure 4:**
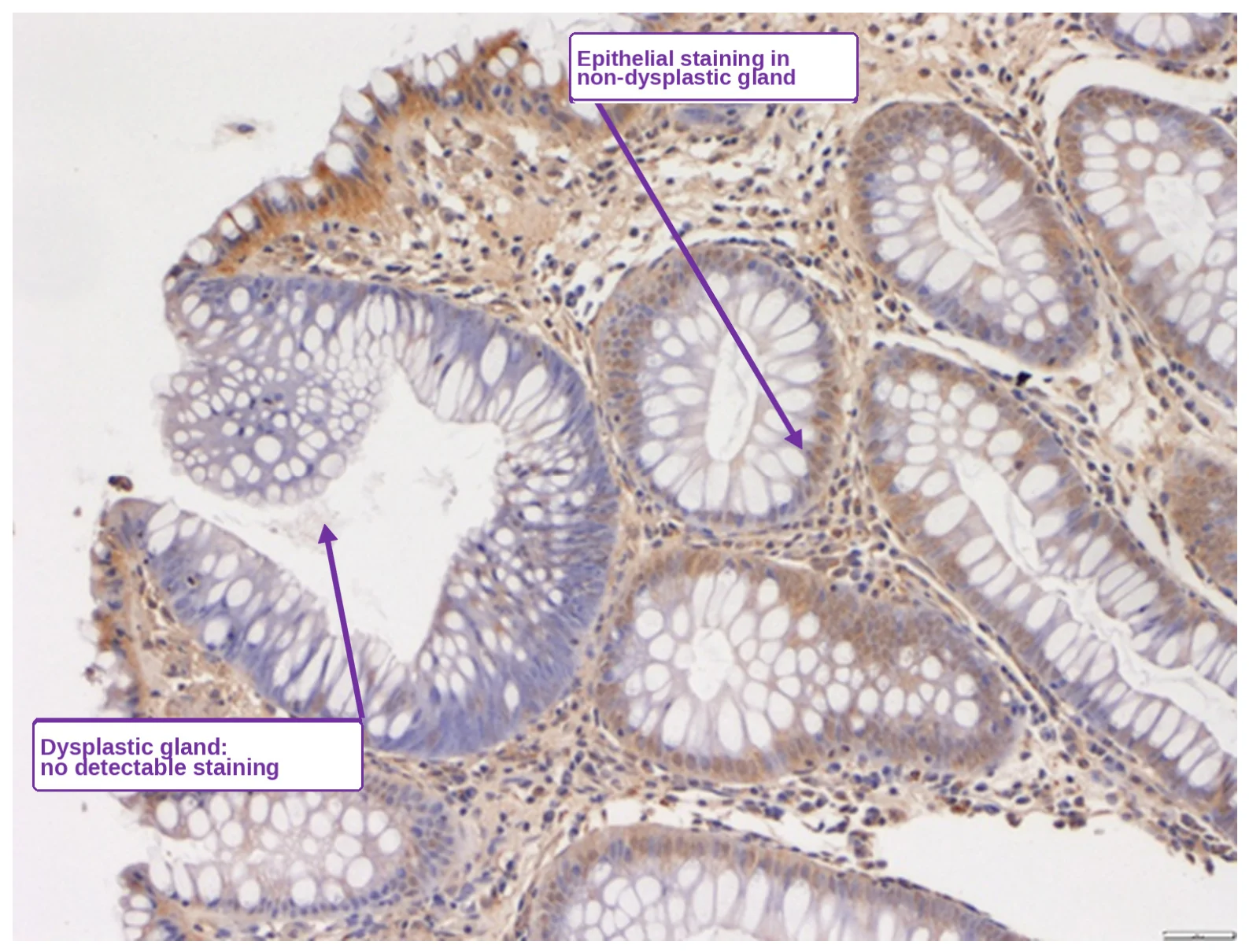
Serrated lesion with dysplasia showing chemerin immunoreactivity in non-dysplastic glandular epithelium and no detectable staining in dysplastic glands. Purple arrows identify epithelial staining in a non-dysplastic gland and a dysplastic gland with no detectable staining (Magnification 100×).

**Figure 5:**
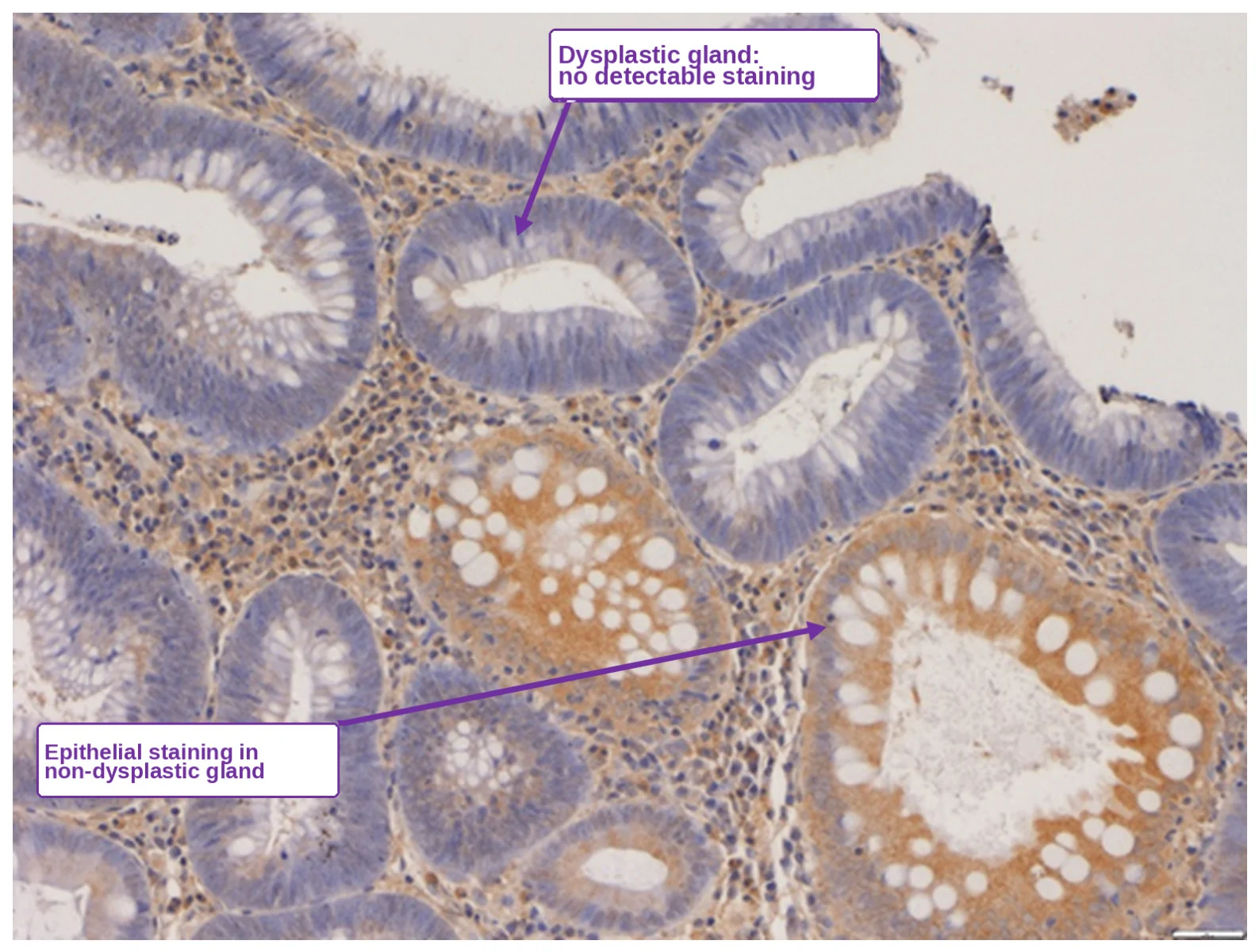
Serrated lesion with dysplasia showing chemerin immunoreactivity in non-dysplastic glandular epithelium and no detectable staining in dysplastic glands. Purple arrows identify epithelial staining in a non-dysplastic gland and a dysplastic gland with no detectable staining (Magnification 100×).

Immunohistochemical analysis did not demonstrate chemerin staining in colorectal adenocarcinoma tissue or conventional adenomatous polyps. In contrast, chemerin immunoreactivity was observed in all available hyperplastic/serrated lesions, with epithelial cytoplasmic and stromal staining [Figure 2 and Figure 3, Table 5]. In lesions containing dysplasia, staining was present in the non-dysplastic glandular epithelium and stroma but was not detected in dysplastic glands [Figure 4 and Figure 5]. Because staining was assessed qualitatively and no semi-quantitative score or independent duplicate pathology review was available, the tissue findings should be reported as preliminary descriptive observations. Exact 95% confidence intervals for the proportion with detectable staining were 0–8.6% for CRC, 0–21.8% for conventional adenomas, and 47.8–100% for hyperplastic/serrated lesions. Thus, the absence of staining in CRC and adenomas argues against frequent detectable chemerin expression under the assay conditions used, while not excluding uncommon expression.

## 4. Discussion

The present exploratory case–control study did not identify significant differences in serum chemerin concentrations between patients with CRC, patients with colorectal polyps and colonoscopy-negative controls in unadjusted pairwise comparisons. This finding does not support serum chemerin as a standalone discriminatory biomarker for colorectal neoplasia in this cohort, but it should not be interpreted as definitive evidence of an absence of association because adjusted modelling was not performed. The study also generated a preliminary tissue observation: chemerin immunoreactivity was detected in all available hyperplastic/serrated lesions, in non-dysplastic epithelial and/or stromal compartments where dysplasia was present, but not in conventional adenomatous polyps or colorectal adenocarcinoma. Because the tissue assessment was qualitative and the hyperplastic/serrated subgroup was small, this finding should be regarded as hypothesis-generating only. Accordingly, the null tissue findings should be interpreted as evidence against chemerin being a commonly detectable tissue marker in CRC or conventional adenoma under the assay conditions used, rather than being dismissed solely as a consequence of sample size.

The null circulating biomarker finding is clinically important because chemerin is strongly linked to metabolic regulation, inflammation, adiposity, glucose status and blood pressure [11,12,13]. These biological properties make chemerin a plausible candidate marker, but also create substantial potential for confounding. In the present study, chemerin was associated with metabolic variables in controls, particularly glucose status, hypertension and body weight. Therefore, the absence of multivariable adjustment is a central limitation. The data cannot exclude a modest disease-related signal masked by metabolic heterogeneity, nor can they prove that chemerin is unrelated to colorectal neoplasia. They show only that unadjusted serum chemerin did not discriminate the study groups in this sample. Consequently, the nominal subgroup *p*-values cannot be interpreted as independent disease effects: age, adiposity, glycaemic status, blood pressure and medication use may account for or obscure the observed associations.

The numerically lower chemerin concentration in the polyp group was unexpected, particularly because tissue immunoreactivity was observed in hyperplastic/serrated lesions. Several explanations are possible. First, the polyp cohort was small and histologically heterogeneous. Second, only a minority of polyp cases were hyperplastic/serrated, whereas the circulating analysis pooled all polyp subtypes. Third, local tissue immunoreactivity in small mucosal lesions would not necessarily translate into higher systemic serum concentrations. Fourth, residual metabolic confounding may dominate any lesion-specific signal. For these reasons, the discordance between serum and tissue findings should be presented as a reason for further study rather than as evidence of a defined biological pathway.

Previous studies have reported inconsistent relationships between chemerin and CRC. Eichelmann et al. reported associations between circulating chemerin and CRC risk, with possible differences by sex and tumour location [17]. Erdogan et al. found higher chemerin concentrations in CRC than controls in a small cohort [18], and Alkady et al. reported associations with nodal metastasis and TNM stage [19]. The present results differ from those reports. The discrepancy may reflect differences in cohort composition, tumour stage distribution, tumour location, metabolic phenotype, sex distribution, assay conditions and statistical adjustment. The current cohort was not powered for sex-stratified, stage-stratified or location-stratified inference.

The tissue findings require particular caution. Qualitative immunohistochemistry can identify whether staining is present, but it cannot quantify intensity, proportion of positive cells, stromal versus epithelial distribution or interobserver reproducibility. A future confirmatory study should predefine the pathology categories, include conventional adenomas and serrated lesions as separate groups, use semi-quantitative scoring such as an H-score or an intensity-proportion score, include independent blinded observers, and report interobserver agreement. Without these steps, the current tissue observations cannot support claims about chemerin as a tissue biomarker. Prospective validation would require a pre-specified sample size calculation, standardised fasting and sample-processing procedures, metabolically characterised or matched controls, multivariable modelling, prospective pathology classification, quantitative immunohistochemical scoring, and the independent external validation of discrimination and calibration.

The study should therefore be positioned conservatively. Its strength is that it reports a negative biomarker evaluation rather than converting a null result into a discovery claim. Its main contribution is to show that unadjusted serum chemerin did not separate CRC, colorectal polyp and control groups and that qualitative staining was not detected in CRC or conventional adenomas under the assay conditions used, while raising a limited tissue-level question regarding hyperplastic/serrated lesions. This framing is more defensible than a broad claim of biomarker discovery.

### Limitations

This study has several important limitations. The sample size was modest, particularly for subgroup analyses by tumour grade, tumour stage, polyp number, glucose status, hypertension status and tissue phenotype. No formal a priori sample size calculation was performed, and the study should therefore be regarded as exploratory. The groups were imbalanced for age and BMI, and multivariable adjustment was not performed. No correction for multiple comparisons was applied, increasing the risk of false-positive findings in exploratory analyses; future confirmatory studies should consider appropriate multiplicity adjustment, such as false discovery rate control [22]. The polyp group was small, and subtype-specific dysplasia mapping was incomplete. Chemerin immunohistochemistry was qualitative only, without semi-quantitative scoring, blinded duplicate assessment or interobserver agreement; the original pathologist was no longer available to perform retrospective scoring. Participant selection and colonoscopy indication were not fully characterised at the individual level, although the cohort was drawn from symptomatic or clinically indicated colonoscopy rather than screening. Smoking status, detailed diabetes status, subtype-specific dysplasia mapping and outlier/sensitivity information were unavailable. These limitations preclude definitive conclusions regarding chemerin as either a circulating or tissue biomarker. Detailed pre-analytical serum-handling information was unavailable for the present analysis, which may have introduced measurement variability and limits reproducibility. The absence of tissue staining should not be attributed simply to sample size: with 0/41 CRC specimens and 0/15 conventional adenomas, the exact upper 95% confidence limits for positivity were 8.6% and 21.8%, respectively. These data argue against frequent expression but cannot exclude uncommon positivity or limitations of the qualitative assay.

## 5. Conclusions

In this exploratory case–control study, serum chemerin concentrations did not significantly differ between patients with CRC, patients with colorectal polyps and colonoscopy-negative controls, and therefore did not support chemerin as a standalone circulating biomarker for colorectal neoplasia. The absence of detectable chemerin immunoreactivity in 41 CRC specimens and 15 conventional adenomas argues against chemerin as a commonly expressed tissue biomarker in these lesions under the assay conditions used. Immunoreactivity in non-dysplastic epithelial and/or stromal compartments of five hyperplastic/serrated lesions, with no detectable staining in dysplastic glands, remains preliminary and should not be interpreted as validation; confirmation would require prospective, adequately powered studies with standardised pre-analytics, detailed pathological classification, semi-quantitative scoring, blinded assessment and multivariable adjustment.

## Figures and Tables

**Table 1:** Baseline characteristics of the study cohort.

Variable	CRC (*n* = 41)	Polyp Group (*n* = 20)	Controls (*n* = 29)
Age, years, median (IQR)	72.0 (63.0–78.0)	56.5 (51.0–61.0)	62.0 (53.75–67.25)
Sex, female/male	18/23	12/8	13/16
Body weight, kg, median (IQR)	75.5 (70.00–85.50)	70.0 (66.50–81.50)	79.0 (68.75–89.75)
BMI, kg/m^2^, median (IQR)	27.4 (25.02–29.30)	25.5 (23.87–26.64)	28.9 (25.38–31.11)
Systolic blood pressure, mmHg, median (IQR)	135.0 (120.00–146.25)	140.0 (126.25–150.00)	135.0 (120.00–150.00)
Diastolic blood pressure, mmHg, median (IQR)	80.0 (70.00–85.00)	82.5 (75.00–90.00)	80.0 (70.00–90.00)
Glucose, mg/dL, median (IQR)	95.0 (85.00–105.50)	92.0 (88.00–96.00)	93.0 (87.00–105.00)
Hypertension, *n* (%)	27 (65.9)	12 (60.0)	19 (65.5)
Antihypertensive treatment, *n* (%)	27 (65.9)	12 (60.0)	19 (65.5)
Hyperglycaemia/diabetes status, *n* (%)	14 (34.1)	5 (25.0)	9 (31.0)
Statin or lipid-lowering therapy, *n* (%)	11 (26.8)	4 (20.0)	4 (13.8)

Abbreviations: BMI, body mass index; CRC, colorectal cancer; IQR, interquartile range. Antihypertensive therapy primarily included ACE inhibitors, sartans, diuretics and beta-blockers. Lipid-lowering therapy primarily included rosuvastatin, atorvastatin and fibrates.

**Table 2:** Clinicopathological characteristics of the colorectal cancer group (*n* = 41).

Characteristic	Category	*n*	%
Anatomical location	Rectum	12	29.3
	Sigmoid colon	15	36.6
	Descending colon	1	2.4
	Transverse colon	3	7.3
	Ascending colon	5	12.2
	Cecum	5	12.2
TNM stage (AJCC 8th edition) [21]	0/TisN0M0	0	0.0
	I/T1N0M0 + T2N0M0	9	22.0
	II/T3N0M0 + T4N0M0	19	46.3
	III/Any T, N1-2, M0	11	26.8
	IV/Any T, any N, M1 (pT4bN2bM1; pT4aN1cM1)	2	4.9

Percentages are calculated using the colorectal cancer cohort as the denominator. TNM classification is reported according to the 8th edition of the AJCC system; the M category was based on imaging tests.

**Table 3:** Clinicopathological characteristics of colorectal polyps included in the study (*n* = 20).

Characteristic	Category	*n* (%)	Size Range, mm
Anatomical location	Rectum	5 (25.0)	
	Sigmoid colon	7 (35.0)	
	Descending colon	2 (10.0)	
	Transverse colon	2 (10.0)	
	Ascending colon	3 (15.0)	
	Cecum	1 (5.0)	
Histological type	Adenomatous polyp	15 (75.0)	3–40
	Tubular adenoma	9 (60.0 of adenomas)	3–20
	Tubulovillous adenoma	6 (40.0 of adenomas)	15–40
	Villous adenoma	0	
	Hyperplastic/serrated lesion	5 (25.0)	3–15
	Hyperplastic polyp	2 (40.0 of serrated/hyperplastic lesions)	5–15
	Sessile serrated lesion	1 (20.0 of serrated/hyperplastic lesions)	10
	Traditional serrated adenoma	0	
	Serrated lesion with dysplasia	2 (40.0 of serrated/hyperplastic lesions)	8–15

Percentages are calculated using the total polyp cohort unless otherwise stated.

**Table 4:** Serum chemerin concentrations in patients with colorectal cancer, patients with colorectal polyps and controls.

Variable	CRC (*n* = 41)	Polyp (*n* = 20)	Control (*n* = 29)	CRC vs. Polyp *p*	CRC vs. Control *p*	Polyp vs. Control *p*
Chemerin, ng/mL, median (IQR)	144.0 (40.27–190.92)	97.4 (29.95–183.70)	135.7 (59.90–155.35)	0.24	0.41	0.94

Abbreviations: CRC, colorectal cancer; IQR, interquartile range. Pairwise comparisons used Mann–Whitney U tests and were interpreted as exploratory.

**Table 5:** Chemerin immunohistochemistry by tissue category.

Tissue Category	*n* Tested	*n* Positive	Positive/Total	Staining Pattern
Colorectal adenocarcinoma tissue	41	0	0/41	No staining detected
Conventional adenomatous polyp tissue	15	0	0/15	No staining detected
Hyperplastic/serrated lesion tissue	5	5	5/5	Epithelial cytoplasmic and stromal staining; absent in dysplastic glands

## Data Availability

The data supporting the findings of this study are available from the corresponding author upon reasonable request.
